# Contributors to suicidality in rural communities: beyond the effects of depression

**DOI:** 10.1186/1471-244X-12-105

**Published:** 2012-08-08

**Authors:** Tonelle E Handley, Kerry J Inder, Frances J Kay-Lambkin, Helen J Stain, Michael Fitzgerald, Terry J Lewin, John R Attia, Brian J Kelly

**Affiliations:** 1 Priority Research Centre for Translational Neuroscience and Mental Health, University of Newcastle, Newcastle, Australia; 2 Hunter Medical Research Institute, Locked Bag 1, Hunter Region Mail Centre, Newcastle, Australia; 3 National Drug and Alcohol Research Centre, University of New South Wales, Sydney, Australia; 4 Centre for Rural and Remote Mental Health, University of Newcastle, Orange, NSW, Australia; 5 Centre for Clinical Epidemiology and Biostatistics, University of Newcastle, Newcastle, Australia; 6 Schizophrenia Research Institute, Sydney, Australia; 7 Department of Medicine, John Hunter Hospital, Locked Bag 1, Hunter Region Mail Centre, Newcastle, Australia

## Abstract

**Background:**

Rural populations experience a higher suicide rate than urban areas despite their comparable prevalence of depression. This suggests the identification of additional contributors is necessary to improve our understanding of suicide risk in rural regions. Investigating the independent contribution of depression, and the impact of co-existing psychiatric disorders, to suicidal ideation and suicide attempts in a rural community sample may provide clarification of the role of depression in rural suicidality.

**Methods:**

618 participants in the Australian Rural Mental Health Study completed the Composite International Diagnostic Interview, providing assessment of lifetime suicidal ideation and attempts, affective disorders, anxiety disorders and substance-use disorders. Logistic regression analyses explored the independent contribution of depression and additional diagnoses to suicidality. A receiver operating characteristic (ROC) analysis was performed to illustrate the benefit of assessing secondary psychiatric diagnoses when determining suicide risk.

**Results:**

Diagnostic criteria for lifetime depressive disorder were met by 28% (174) of the sample; 25% (154) had a history of suicidal ideation. Overall, 41% (63) of participants with lifetime suicidal ideation and 34% (16) of participants with a lifetime suicide attempt had no history of depression. When lifetime depression was controlled for, suicidal ***ideation*** was predicted by younger age, being currently unmarried, and lifetime anxiety or post-traumatic stress disorder. In addition to depression, suicide ***attempts*** were predicted by lifetime anxiety and drug use disorders, as well as younger age; being currently married and employed were significant protective factors. The presence of comorbid depression and PTSD significantly increased the odds of reporting a suicide attempt above either of these conditions independently.

**Conclusions:**

While depression contributes significantly to suicidal ideation, and is a key risk factor for suicide attempts, other clinical and demographic factors played an important role in this rural sample. Consideration of the contribution of factors such as substance use and anxiety disorders to suicidal ideation and behaviours may improve our ability to identify individuals at risk of suicide. Acknowledging the contribution of these factors to rural suicide may also result in more effective approaches for the identification and treatment of at-risk individuals.

## Background

Prior suicidal ideation or suicide attempts are the primary risk factors for future suicidal behaviours [1,2]. There is a strong relationship between suicidality and depression, with affective disorders contributing to the incidence of suicidal ideation, plans and attempts [3]. The frequent co-occurrence of these conditions has caused suicidality to become largely conceptualised as a symptom of depression, and as such is included as a diagnostic criterion for Major Depressive Episode in the DSM-IV [4], as well as appearing in a range of instruments for measuring depression (e.g. Beck Depression Inventory, Composite International Diagnostic Interview, Patient Health Questionnaire, Hamilton Rating Scale for Depression) [5]. Although suicidality also appears in the DSM-IV diagnostic criteria for Borderline Personality Disorder, there is a greater focus on recurrent and severe suicidal behaviours and therefore milder or transient suicidality may not be considered relevant. Evidence shows that in the majority of cases, suicide attempts are preceded by more minor expressions of suicidality such as life weariness or death wishes [1]. Therefore the exclusion of “mild” suicidality from diagnostic criteria may prevent adequate attention being directed towards individuals at risk of future suicidal behaviour.

Emerging evidence suggests that considering suicidality primarily as a component of a depressive disorder may lead to suicide risk being underestimated or overlooked in clinical settings [6]. There is preliminary evidence for a dual-factor model of depression and suicidality as related but independent constructs [5], with support for this model evident in a range of previous community studies. For example, a recent international study found that across a variety of countries and urban/rural localities, suicidal ideation occurred more frequently than depression [7]. Similarly, it is a consistent finding that males have a significantly higher suicide rate than females [8], despite rates of depression among males generally being found to be lower than their female counterparts [9]. Evidence from psychological autopsy studies shows that approximately one-third of suicide victims do not have symptoms indicative of major depression at their time of death [10], suggesting the importance of other contributory factors in a substantial proportion of those who die by suicide.

Suicidality occurs in individuals with a range of psychiatric disorders [11]. Up to 63% of suicide victims experience substance use disorders, including both drugs and alcohol [12], while over 70% of those who make a suicide attempt have at least one anxiety disorder [13]. Additionally, approximately 80% of individuals with bipolar disorder experience suicidal ideation or a suicide attempt during their lifetime [14], while individuals with schizophrenia are also at a significantly increased risk of suicide [15]. Comorbidity also plays a vital role in suicidality, greatly increasing the likelihood of suicidal behaviours [16-18]. In addition, psychological autopsy studies show that approximately 10% of those who take their own life did not meet criteria for any psychiatric diagnosis [19]. These findings strongly suggest that it would be beneficial to assess suicidality in a range of psychiatric disorders, rather than restricting these assessments primarily to depressive conditions. Similarly, the small proportion of individuals whose suicidality occurs in the absence of any psychiatric condition implies that the identification of additional non-psychological risk factors is necessary.

The conceptualisation of suicidality predominantly as a symptom of depression has restricted research exploring the relationship between these variables beyond their common co-occurrence [5]. As a result, there are few existing studies exploring suicidality as independent from depression in large-scale community samples [20]. This research may be especially valuable in rural populations as suicide rates are generally higher in rural areas [21], yet the prevalence of mood disorders is often equivalent to that observed in urban regions [22]. Additionally, while the concept of suicidality and depression as separable states has been explored in one Australian study [5], this study used a predominantly urban sample; the generalisability to rural populations has not yet been explored. Identifying the independent contribution of depression to suicidality in rural areas will allow an estimation of the extent to which additional factors may influence rural suicide.

This study aims to explore the prevalence of suicidal thoughts and behaviours among individuals with and without a history of depressive illness in a non-metropolitan community sample. It investigates both the independent contribution of depression to suicidality, and the influence of additional psychiatric diagnoses, including anxiety and substance use. Based on previous research conducted in urban and rural populations [5,7], it is hypothesised that depression will have a strong association with suicidality, yet we anticipate that additional predictors will contribute to the presence of suicidality in both individuals with and without a depressive disorder. The information obtained from this study may be useful for the future identification and treatment of individuals at risk of suicidal behaviour who may not be recognised under current screening procedures.

## Methods

### Participants

Data were obtained from the baseline phase of the Australian Rural Mental Health Study (ARMHS), a longitudinal population-based study exploring determinants of mental health in rural and remote communities, with a focus on the influence of social factors (see [23] for a detailed description). Baseline data were collected between 2006 and 2009. The sample consisted of New South Wales (NSW) residents aged 18 or over, who were randomly selected from the Australian Electoral Roll and resided in one of 60 Local Government Areas (LGAs) from the Greater Western, Hunter New England, or North Coast rural health service regions of NSW. These areas cover approximately 70% of the geographic region of non-metropolitan NSW. Metropolitan areas, including capital cities and other urban centres with populations greater than 100,000, were excluded.

### Measures

#### Demographics

Age, gender, marital status, education and employment status were assessed by single-item questions via an initial postal survey. This postal survey also included the Kessler-10 (K10) psychological distress scale, which was used to select participants for telephone interview as described below.

#### The Composite International Diagnostic Interview

The World Health Organisation Composite International Diagnostic Interview (CIDI) is a standardised diagnostic interview used to assess the presence of a range of mental disorders according to both Diagnostic and Statistical Manual (DSM-IV) and International Classification of Diseases (ICD-10) criteria [24]. Participants were selected for CIDI interview based on their K10 psychological distress score [25]; interviews were offered to 100% of those with a high-range score (25+), 75% of those with a moderate-range score (16–24), and one-sixth of those scoring in the low range (10–15). The CIDI has been shown to have excellent inter-rater reliability, and good validity and test-retest reliability, and is an acceptable method to determine lifetime diagnoses [26], using both face-to-face and telephone delivery [27-30]. This interview was used to determine the presence of suicidal ideation at any time during the participants’ life (“have you ever seriously thought about committing suicide?”). Participants who endorsed suicidal ideation at any time during their life were subsequently asked about their history of suicide attempts. If participants had no lifetime suicidal ideation they were not asked about lifetime suicide attempts; for these participants it was assumed that they had never attempted suicide. The CIDI suicidality section also includes questions about participants’ age at their first and most recent thoughts of suicide and/or suicide attempts, the number of suicide attempts they have made, and the severity of their lethal intent.

The CIDI was also used to assess a lifetime diagnosis of any depressive disorder, including unipolar and bipolar major depression, as well as the subthreshold conditions of dysthymia and minor depression (“depressive disorder”). An inclusive approach was utilised whereby the standard exclusion criteria for bereavement and medical attribution were not applied [31]; this allowed for exploration of the effects of depression symptoms regardless of their cause. The CIDI interview generated diagnoses of generalised anxiety disorder, social phobia, agoraphobia, panic attack and panic disorder (“anxiety disorder”); post-traumatic stress disorder (PTSD); alcohol abuse or dependence (“alcohol use disorder”); and drug abuse or dependence (“drug use disorder”). Due to evidence indicating a strong relationship between PTSD and suicidality [32], this condition was examined independently, rather than being collapsed into “anxiety disorders.”

### Consent and ethical approval

Introductory letters were sent out to participants prior to dispatch of postal surveys, and information sheets and consent forms were included with each survey. These documents contained a description of both the postal survey and CIDI interview. Written informed consent was therefore obtained from each participant with the return of their postal survey. For CIDI interviews, verbal consent was re-confirmed over the phone at the time of interview scheduling. This research was conducted in compliance with the Helsinki Declaration; ethical approval was obtained from the Human Research Ethics Committees of the University of Newcastle (reference: H-145-1105a) and University of Sydney (reference: 13069).

### Statistical analysis

Data analysis was conducted using PASW (version 18; PASW, Chicago, IL, USA) and Stata (release 11; College Station, TX: StataCorp LP). Analyses were initially stratified by depression status; that is, the determinants of suicidal ideation and suicide attempts were explored independently for individuals with and without a history of depression. Univariate analyses were performed on all data; all variables were analysed using a chi-squared test. Odds ratios were used to represent the degree of influence of these variables on suicidality. An interaction was then calculated to determine differential effects of predictors between these groups.

Hierarchical logistic regressions were used to predict suicidal ideation and suicide attempts respectively; predictor variables were entered independently at step 1, while their interaction with depression was entered at step 2. In order to determine the potential impact of our selection process (i.e. selecting participants based on K10 scores), these analyses were also conducted with data back-weighted to be representative of the entire ARMHS sample, for comparative purposes.

To illustrate the relative contributions of depression and other factors to suicidality, two ROC curves were constructed with suicidal ideation and suicide attempt status as the respective outcomes. Depression status was plotted alone, and in conjunction with the full range of other psychological conditions and demographics. The area under the curve (AUC) for each of these models was compared to explore the additional predictive power obtained by considering secondary disorders when assessing risk of suicidality.

## Results

Of the 2639 ARMHS participants recruited, 867 were selected for CIDI interview based on their K10 score, and 230 (27%) participants declined, therefore 637 (73%) were interviewed. The mean age of participants who declined the CIDI (M = 50.2, SD = 16.2) was younger than those who accepted (M = 55.3, SD = 13.8, *F*(1, 866) = 21.3, *p* < .001); no other demographic differences were observed between these groups. Due to interviewer error, five participants were not administered the suicidality section, and a further 14 participants gave a “don’t know” response when asked about their history of suicidal ideation, resulting in data for 618 participants. The mean age was 55.2 years; two hundred and forty-four (39%) participants were male. One hundred and seventy-four (28%) participants met criteria for a depressive disorder. Of these, ten had minor depression, five experienced dysthymia only, 24 met criteria for both dysthymia and major depression (“double depression”), 112 experienced lifetime major depression only, and 23 had lifetime bipolar disorder. When the suicidality criterion was removed, all participants still met criteria for these diagnoses, therefore the presence of suicidality as a diagnostic criterion is not perceived to have confounded our results. Of the remaining sample, 250 (40.5%) indicated that they had ever experienced an episode of feeling “sad, empty or depressed” that did not last for longer than several days, while 194 participants (31.4%) reported no lifetime symptoms of depression.

**Figure 1:**
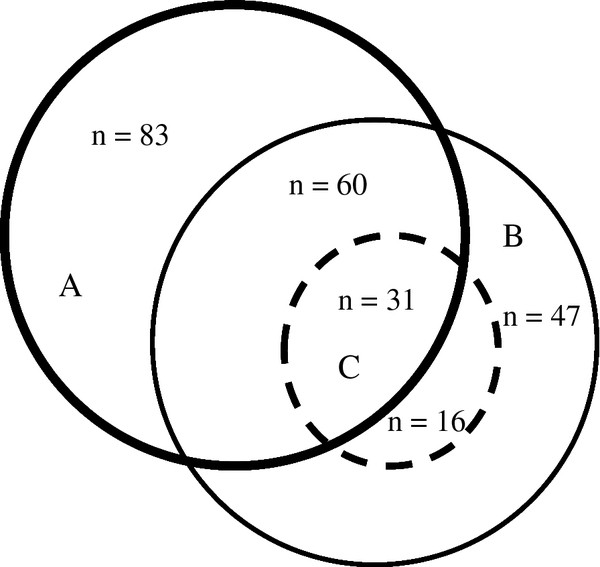
Relationship between (A) depressive disorder, (B) suicidal ideation and (C) suicide attempt.

One hundred and fifty-four participants (25%) indicated a history of suicidal ideation, and forty-seven (31%) of these participants reported having made at least one suicide attempt during their lifetime. The relationship between depression, suicidal ideation and suicide attempts is shown in Figure 1.

### Suicidal ideation

#### Characteristics of participants with lifetime suicidal ideation

The mean age of individuals with lifetime suicidal ideation was 50.9 years, with 53 (34%) of this sample being male. Of these individuals, 91 (58%) met criteria for a lifetime diagnosis of depression; this was not affected when the DSM-IV suicidality item (item 9) was removed. There was a significant univariate association between lifetime suicidal ideation and lifetime depressive disorder, with depressive disorder increasing the odds of reporting suicidal ideation six-fold (OR = 6.6, 95% CI 4.5-9.9, *p* < .001). The presence of suicidal ideation differed significantly by type of depressive disorder (i.e. minor depression, dysthymia, major depression, double depression or bipolar), *χ*_(4)_^2^ = 13.0, *p* = .011; major depression (either unipolar or bipolar) was a greater risk factor for suicidal ideation than dysthymia or minor depression. Nineteen (12%) individuals with lifetime suicidal ideation did not meet diagnostic criteria for any disorder. These participants were significantly older (M = 58.0, SD = 13.8) than participants whose ideation was in the context of a psychiatric condition (M = 49.9, SD = 13.1, *F*(1,153) = 6.2, *p* = .014); no other demographic differences were observed.

Of the 154 participants with a lifetime history of suicidal ideation, 63 (42%) did not have a lifetime history of depressive disorder. As shown in Table 1, individuals aged 65 and over, and individuals who were currently married, were less likely to experience thoughts of suicide. On a univariate basis, the odds of experiencing suicidal ideation increased with a lifetime history of depressive illness, drug use disorder, alcohol use disorder, PTSD or other anxiety disorder. The strongest univariate relationship was seen for depressive disorder (OR = 6.6) A lifetime history of more than one diagnosis was also predictive of suicidal ideation (OR = 5.8, 95% CI 3.9-8.5, *p* < .001). In the univariate analyses, there were no significant interactions between lifetime depressive disorder and any predictors.

**Table 1:** Relationships between demographic/clinical characteristics and lifetime suicidal ideation rates: overall and by lifetime depression status

**Demographic/clinical characteristic**		**With depression**	**Without depression**	**Overall**	**Overall**			
		**n cases/total (%)**	**n cases/total (%)**	**n cases/total (%)**	**Unadjusted**		**Adjusted**	
					**OR**	**95% CI**	**AOR**	**95% CI**
*Demographic characteristics*								
Age	Under 45	27/43 (63)	20/92 (22)	47/135 (35)	3.1**	1.8-5.5	3.6**	1.6-8.1
	45-64	52/104 (50)	33/229 (14)	85/333 (26)	2.0**	1.2-3.3	2.2*	1.1-4.3
	65+	12/27 (44)	10/123 (8.1)	22/150 (15)				
Gender	Male	31/54 (57)	22/190 (12)	53/244 (22)	0.75	0.51-1.1	0.88	0.54-1.4
	Female	60/120 (50)	41/254 (16)	101/374 (27)				
Marital status	Married/de facto	45/94 (49)	35/338 (10)	80/432 (19)	0.34**	0.23-0.50	0.46**	0.29-0.74
	Not married	45/78 (58)	28/105 (27)	73/183 (40)				
Education	High school or other	60/122 (49)	44/297 (15)	104/419 (25)	1.2	0.75-1.8	1.0	0.61-1.8
	Didn’t finish school	20/38 (53)	15/120 (13)	35/158 (22)				
Employment status	Employed	50/102 (49)	36/255 (14)	86/357 (24)	0.90	0.62-1.3	0.53*	0.32-0.89
	Not in workforce	40/69 (58)	27/188 (14)	67/257 (26)				
*Lifetime DSM-IV diagnoses*								
Depressive disorder	Yes	91/174 (52)	-	91/174 (52)	6.6**	4.4-9.9	3.0**	2.0-5.3
	No	-	63/444 (14)	63/444 (14)				
Alcohol disorder	Yes	36/52 (69)	15/82 (18)	51/134 (38)	2.8**	1.5-3.4	1.6	0.90-2.7
	No	55/122 (45)	48/362 (13)	103/484 (21)				
Drug use disorder	Yes	15/21 (71)	7 /15 (47)	22/36 (61)	5.4**	2.7-11	2.1	0.82-5.2
	No	76/153 (50)	56/429 (13)	132/582 (23)				
Anxiety disorder	Yes	69/120 (58)	30/121 (25)	99/241 (41)	4.1**	2.8-6.0	2.0**	1.3-4.3
	No	22/54 (41)	33/323 (10)	55/377 (15)				
PTSD	Yes	39/59 (66)	13/41 (33)	52/100 (55)	4.4**	2.8-6.9	2.4**	1.4-4.4
	No	52/115 (45)	50/403 (12)	102/518 (20)				

Note: Overall Odds Ratios (OR) and Confidence Intervals (CI) from unadjusted and adjusted (multivariate).

logistic regressions: *p < .05, **p < .01.

As can be seen in Table 1, the multivariate analysis revealed a range of significant predictors for lifetime suicidal ideation. Participants aged 65 and over were less likely to experience thoughts of suicide than participants aged under 45 and those aged 45–64, and being currently married remained a significant protective factor. Similarly, being currently employed was associated with lower lifetime suicidal ideation. A lifetime history of depressive disorder, anxiety disorder and PTSD were all significantly associated with and increased odds of experiencing lifetime suicidal ideation. As in the univariate results, depressive disorder was the strongest correlate of suicidal ideation when other factors were controlled for (OR = 3.0). The multivariate model also revealed two significant interactions, for marital status and drug use disorder. However, when data were back-weighted to be representative of the entire ARMHS sample, these interactions were no longer significant, indicating that these findings may have been the result of selection effects in our data. In the back-weighted data set, lifetime drug use disorder was a significant risk factor (OR = 4.3, 95% CI 1.3-15), and male gender was protective (OR = 0.48, 95% CI 0.26-0.90); all other results replicated those found in our original analysis.

**Figure 2:**
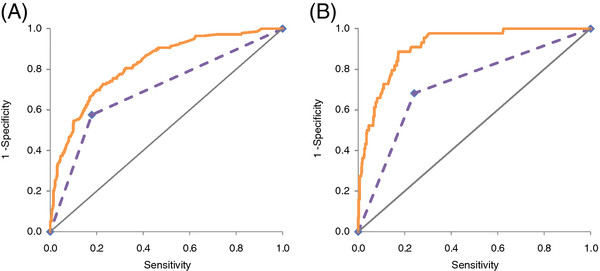
ROC curve comparing accuracy of depressive disorders alone (broken line) versus in combination with additional diagnoses and demographic variables (solid line), in predicting (A) lifetime suicidal ideation, and (B) lifetime suicide attempts.

As shown in Figure 2a, the ROC curve for suicidal ideation was originally computed for depressive disorders alone (AUC = 0.70, 95% CI 0.66-0.75). Adding demographic characteristics and additional diagnoses (AUC = 0.81, 95% CI 0.77-0.85) significantly improved the AUC (*χ*_(1)_^2^ = 38.7, *p* < .001).

### Suicide attempts

#### Characteristics of participants with a lifetime suicide attempt

Of the 47 participants who had attempted suicide in their lifetime, the mean age was 46.9 years; seventeen participants (36%) were male. Sixteen (34%) individuals who had made a lifetime suicide attempt did not have a lifetime diagnosis of depression. However all participants with a lifetime suicide attempt did meet criteria for at least one psychiatric disorder. All participants with a history of suicide attempts made their attempts while residing in a rural area.

As shown in Table 2, at a univariate level participants aged 65 and over, and those who were currently married were significantly less likely to report a lifetime suicide attempt. They were also more likely to have a lifetime depressive, alcohol use, drug use or anxiety disorder, or lifetime PTSD. The presence of two or more comorbid disorders was also a significant univariate risk factor (OR = 5.9, 95% CI 3.1-11, *p* < .001). The logistic regression revealed that at a multivariate level, being aged 65 and over reduced the likelihood of reporting a lifetime suicide attempt compared to those aged under 45, while being currently married and employed were significant protective factors. The presence of a lifetime depressive, drug use or anxiety disorder each significantly increased an individual’s likelihood of reporting a suicide attempt. While depression was a significant univariate correlate, this effect was only marginal in the regression (*p* = .05). A significant interaction was also observed between depression and PTSD; PTSD was a significantly greater risk factor for those with (OR = 15, 95% CI 6.8-31) compared to without (OR = 0.71, 95% CI 0.09-5.5) lifetime depression (see Additional file 1). This represents an interaction OR of 7.1, i.e. suicide attempts when both factors are present are 7 fold higher than might be expected based on the effects on depression and PTSD separately.

**Table 2:** Relationships between demographic/clinical characteristics and lifetime suicide attempt: overall and by lifetime depression status

**Demographic/clinical characteristic**		**With depression**	**Without depression**	**Overall**	**Overall**			
		**n cases/total (%)**	**n cases/total (%)**	**n cases/total (%)**	**Unadjusted**		**Adjusted**	
					**OR**	**95% CI**	**AOR**	**95% CI**
*Demographic characteristics*								
Age	Under 45	12/43 (28)	6/92 (6.5)	18/135 (13)	5.6**	1.9-17	4.7*	1.2-18
	45-64	17/104 (16)	8/229 (3.5)	25/333 (7.5)	3.0*	1.0-8.7	2.3	0.64-7.8
	65+	2/27 (7.4)	2/123 (1.6)	4/150 (2.7)				
Gender	Male	10/54 (19)	7/190 (3.7)	17/244 (7.0)	0.86	0.46-1.6	0.94	0.43-2.0
	Female	21/120 (18)	9/254 (3.5)	30/374 (8.0)				
Marital status	Married/de facto	11/94 (12)	8/338 (2.4)	19/432 (4.4)	0.26**	0.14-0.47	0.38**	0.18-0.79
	Not married	20/78 (26)	8/105 (7.6)	28/183 (15)				
Education	High school or other	23/122 (19)	9/297 (3.0)	32/419 (7.6)	1.0	0.50-2.0	1.1	0.45-2.5
	Didn’t finish school	7/38 (18)	5/120 (4.2)	12/158 (7.6)				
Employment status	Employed	17/102 (17)	6/255 (2.4)	23/357 (6.4)	0.67	0.37-1.2	0.32**	0.14-0.71
	Not in workforce	14/69 (20)	10/188 (5.3)	24/257 (9.3)				
*Lifetime DSM-IV diagnoses*								
Depressive disorder	Yes	31/174 (18)	-	31/174 (18)	5.8**	3.1-11	2.2†	0.99-4.8
	No	-	16/444 (3.6)	16/444 (3.6)				
Alcohol disorder	Yes	15/52 (29)	5/82 (6.1)	20/134 (15)	3.0**	1.6-5.5	1.4	0.64-3.3
	No	16/122 (39)	11/362 (3.0)	27/484 (5.6)				
Drug use disorder	Yes	10/21 (48)	2/15 (13)	12/36 (33)	7.8**	3.6-17	4.3**	1.4-12.5
	No	21/153 (14)	14/429 (3.3)	35/582 (6.0)				
Anxiety disorder	Yes	26/120 (22)	13/121 (11)	39/241 (16)	8.9**	4.1-19	5.8**	2.2-15.6
	No	5/54 (9.3)	3/323 (0.93)	8/377 (2.1)				
PTSD	Yes	20/59 (34)	2/41 (4.9)	22/100 (22)	5.6**	3.0-10	1.8	0.83-4.0#
	No	11/115 (9.6)	14/403 (3.5)	25/518 (4.8)				

Note: Overall Odds Ratios (OR) and Confidence Intervals (CI) from unadjusted and adjusted (multivariate).

logistic regressions: †*p* = .05, *p < .05, **p < .01.

# Significant interaction with depression status < .05.

When data were back-weighted, the unweighted results were replicated, with the exception that younger age was no longer a significant predictor.

#### Impact of depression on suicide attempts

Overall, depressive disorder was a significant predictor of a suicide attempt, however a lifetime suicide attempt was significantly more likely within some depressive diagnoses (*p <* .001); participants with major depression (either unipolar or bipolar) were at greater risk than those with minor depression or dysthymia. Among individuals with a history of suicide attempts, the mean number of attempts did not differ between participants with (M = 2.6, SD = 3.2) and without (M = 1.8, SD = 1.3) a history of depression (*p* = .287). These individuals also did not differ in age at their first (*p =* .186) or most recent (*p* = .757) suicide attempt, or in the severity of their attempts (*p* = .729). Of the 31 participants with a history of both depression and suicide attempts, 18 (58%) indicated that their suicide attempt occurred during their worst depressive episode; beyond this, we could not determine whether suicide attempts occurred during or outside of a depressive episode.

For the prediction of suicide attempts, depressive disorders alone resulted in an AUC of 0.72 (95% CI 0.64-0.80). As can be seen in Figure 2b, the addition of the remaining predictors increased the AUC to 0.88 (95% CI 0.84-0.93); this was a significant increase (*χ*_(1)_^2^ = 18.1, *p* < .001).

## Discussion

This study aimed to further explore the recent conceptualisation of depression and suicidality as related yet independent constructs. Our findings revealed that approximately one-third of individuals with lifetime suicide attempts and over one-third of individuals with lifetime suicidal ideation did not have a history of depressive disorder, providing support for the emerging theory of these conditions as separable states. This concurs with previous research conducted in an urban Australian population [5], which found strong evidence for depression and suicidality as independent constructs. This indicates a similarity in psychological mechanisms across geographical areas. On a univariate basis, lifetime depressive disorder was strongly related to suicidal ideation and suicide attempts, producing approximately a six-fold increase in the likelihood of an individual experiencing either of these phenomena.

Importantly, all of our participants who met diagnostic criteria for lifetime depression did so even when the DSM-IV suicidality item (item 9) was removed. Therefore the association observed between depression and suicidal ideation was not solely attributable to the inclusion of suicidal ideation in the symptom criteria for depressive disorder. However we acknowledge that the CIDI focuses on the participants’ most severe lifetime depressive episode, which is likely to explain this finding. That is, as the CIDI focuses on the episode for which the most symptoms were endorsed, removing one of these symptoms may not have had as great an impact on meeting diagnostic criteria as it would have when focusing on a less severe episode.

In both univariate and multivariate analyses, depression was the strongest predictor of suicidal ideation. However, in addition to depressive disorder, a variety of other factors were associated with thoughts of suicide. Significant multivariate effects were observed for both PTSD and anxiety disorder, while being unmarried was also associated with an increased likelihood of experiencing lifetime suicidal ideation. Marital status serves as an important proxy for social support, and our findings emphasise the moderating role of interpersonal relationships for individuals who may be at risk of suicide despite having no depressive history. Therefore, programs aimed to enhance social support among rural communities may be an effective public health approach for the reduction of rural suicide. The finding that younger participants were more likely to endorse lifetime suicidal ideation and attempts has been observed in previous Australian community-based research, both for suicidality [33], and for other psychological conditions such as depression, anxiety, and alcohol use [34,35]. This suggests that there may be a cohort effect, in that psychological conditions may be becoming increasingly common in younger generations, or this may be a survivor effect. Alternatively this may be due to a bias in lifetime recall, since older participants may be less likely to remember suicidal thoughts and behaviours if they occurred many years ago. Consistent with previous evidence [19], we also detected a small proportion of individuals experiencing suicidal ideation in the absence of any psychiatric diagnosis. Indicators of the clinical severity of suicidality (e.g. age at first suicidal thoughts and attempt, number and severity of suicide attempts) did not differ among participants with or without a lifetime diagnosis of depression.

When data were back-weighted to match the entire ARMHS sample, our results for suicidal ideation remained largely unchanged. The back-weighted data continued to support the finding that additional demographic factors and psychological diagnoses may contribute to suicide risk when depression is accounted for.

While a strong univariate relationship was observed between depression and lifetime suicide attempts, the multivariate association was weaker, and reached only marginal significance. Unlike suicidal ideation, depression was not the strongest correlate of lifetime suicide attempts on either a univariate or multivariate basis. This indicates an important additional role of secondary psychiatric diagnoses.

Both lifetime drug use and anxiety disorders showed a strong relationship with lifetime suicide attempts, and are important risk factors. There was also a significant interaction for suicide attempts between depression and PTSD; among individuals with depression, a comorbid diagnosis of PTSD greatly increased the odds of a suicide attempt. An increased awareness of the additional potential for suicidal behaviours among this group is warranted. These results were replicated even when data were back-weighted, indicating that these findings may be applicable to larger populations, and were not skewed by our over-representation of people with elevated psychological distress.

Our ROC analyses revealed that depression alone is somewhat limited in its predictive power for both suicidal ideation and suicide attempts. Our analysis indicated the necessity to assess a wider range of psychological conditions in evaluating suicidality.

The main implications of these findings are likely to apply in treatment settings. At present, the consideration of suicidality primarily as a product of a depressive episode has resulted in it frequently being treated as such. During assessments by clinicians, if no evidence is found for the primary symptoms of depression, assessment of “secondary” symptoms such as suicidality may not be undertaken [6], and therefore these individuals may not be referred for further treatment. Clinicians may feel less equipped to assist individuals who experience thoughts of suicide independently of a depressive illness. Where diagnostic criteria for depression are met, suicidality is generally considered as just one factor contributing to an overall diagnosis, and may not be highlighted as a focus of concern above other, less severe, symptoms [6]. Considering recent findings that suicidality and depression may be relatively independent constructs [5], the assumption that treating the overall depressive state extends to effective treatment of individual symptoms such as suicidality may be somewhat unfounded. In particular, people presenting with comorbid psychiatric conditions (especially PTSD) may require integrated treatments for these conditions in order for their suicide risk to be reduced [36]. Our findings highlight the potential need to consider different risk factors for individuals depending on their depressive history and personal circumstances.

The present research is limited by low participant numbers, which may have restricted our power to detect effects, particularly for the interactions. Due to the restricted sample size, it is possible that the present study underestimates characteristics that may distinguish between individuals who experience suicidality within and outside of the context of a depressive episode. Similarly, we had insufficient numbers of participants with a psychotic disorder to include this as a predictor, despite the likely impact of this condition [15]. Our sample was entirely non-metropolitan, and therefore is not representative of urban residents. Our analyses focused primarily on lifetime diagnoses of psychiatric disorders, which have been found to be subject to inaccuracy of recall [37], therefore our findings may be biased towards the null. Additionally, the use of lifetime diagnoses did not allow us to determine whether depression preceded suicidality in participants who experienced both of these conditions. It is possible that among participants who met criteria for both depression and suicidal thoughts or behaviours during their lifetime, the suicidality may still have occurred in the absence of a depressive episode. Future research utilising a larger sample may enable collection of sufficient data to focus on 12-month diagnoses, which may be of greater relevance. By assuming that participants without suicidal ideation also did not have a lifetime suicide attempt, impulsive attempts were not able to be detected in our analysis. Therefore it is likely that this study underestimates the total number of suicide attempts, and overstates the contribution of depression to such attempts. This may be addressed in future by using a less structured assessment which allows for the exploration of suicide attempts among individuals with no history of ideation. Future research may also benefit from the inclusion of a measure of impulsivity, as this is likely to contribute to suicide attempts among individuals experiencing suicidal ideation [38].

## Conclusions

Although there are limitations to the present findings, the study provides supporting evidence that depression and suicidality, although strongly related, appear to be independent constructs. That is, while depression is a significant predictor of suicidal ideation and attempts, it is not a necessary precondition for suicidality to occur, and the contribution of anxiety and substance use disorders to suicidality may be of similar magnitude. If further support were found for the model of depression and suicidality as independent conditions, this may have significant clinical implications, particularly for suicide prevention strategies that chiefly focus on depression. Our findings suggest that a range of psychological conditions may increase suicide risk in individuals whose suicidality is not in the context of a depressive episode, and that the magnitude of the risk posed by conditions such as PTSD vary depending on the individual’s depressive history. Further research focusing on identifying risk factors within additional disorders, particularly anxiety and drug use, would be of value.

## Competing interests

The authors declare that they have no competing interests.

## Authors’ contributions

TEH drafted manuscript, and contributed to data analysis and interpretation; KJI and FJKL contributed to the concept, analysis and interpretation of the data and revision of the manuscript; HJS contributed to ARMHS study design and development, data collection, and manuscript writing and revisions; MF assisted with study design, statistical analysis and interpretation; TJL contributed to ARMHS study development, study design, statistical analysis and interpretation; JRA assisted with manuscript drafting, data analysis and interpretation; BJK contributed to overall ARHMS study development, funding and implementation, and assisted with data analysis, interpretation, and drafting manuscript. All authors read and approved final draft.

## Pre-publication history

The pre-publication history for this paper can be accessed here:

http://www.biomedcentral.com/1471-244X/12/105/prepub

## Supplementary Material

Additional file 1 Interaction between depression and PTSD status for the prediction of lifetime suicide attempt; odds ratio (95% CI). Click here for file
