# Supplementary material for: Contributors to suicidality in rural communities: beyond the effects of depression
Source: BMC Psychiatry. 2012 Aug 8;12:105. doi: 10.1186/1471-244X-12-105 (PMC3477044; doi:10.1186/1471-244X-12-105)
Supplement: Additional file 1 — Interaction between depression and PTSD status for the prediction of lifetime suicide attempt; odds ratio (95% CI). [file 1471-244X-12-105-S1.doc]

|  | **PTSD** | |
| --- | --- | --- |
|  | Yes | No |
| **Depression** |  |  |
| Yes | 15 (6.8-31) | 2.9 (1.3-6.3) |
| No | 0.71 (0.09-5.5) | - |
